# Porta hepatis lymph nodes on US: not only identify biliary atresia but also predict outcomes after Kasai portoenterostomy surgery

**DOI:** 10.1186/s13244-024-01735-3

**Published:** 2024-06-20

**Authors:** Fengying Ye, Wen Ling, Qiumei Wu, Hong Ma, Zhen Huang, Yifan Fang, Guorong Lyu, Zongjie Weng

**Affiliations:** 1 Department of Medical Ultrasonics, Fujian Maternity and Child Health Hospital, College of Clinical Medicine for Obstetrics & Gynecology and Pediatrics, Fujian Medical University, Fuzhou, China; 2 Department of Pathology, Fujian Maternity and Child Health Hospital, College of Clinical Medicine for Obstetrics & Gynecology and Pediatrics, Fujian Medical University, Fuzhou, China; 3 Department of Pediatric Surgery, Fujian Maternity and Child Health Hospital, College of Clinical Medicine for Obstetrics & Gynecology and Pediatrics, Fujian Medical University, Fuzhou, China; 4 Department of Medical Ultrasonics, the Second Affiliated Hospital of Fujian Medical University, Quanzhou, China; 5 Department of Clinical Medicine, Quanzhou Medical College, Quanzhou, China

**Keywords:** Ultrasound, Lymph node, Diagnosis, Kasai surgery

## Abstract

**Objectives:**

To evaluate the usefulness of porta hepatis lymph nodes (PHLNs) on ultrasonography (US) scans in diagnosing biliary atresia (BA) and predicting the outcomes after Kasai portoenterostomy (KPE) surgery.

**Methods:**

A total of 668 patients from one hospital were enrolled in the study (542 non-BA and 126 BA). The independent and combined diagnostic efficacy of PHLNs, triangular cord (TC) thickness, and gallbladder morphology were assessed by drawing the receiver operating characteristic (ROC) curves and counting the area under the ROC curve (AUC), sensitivity, specificity, positive predictive value (PPV), and negative predictive value (NPV). The US features, histopathological findings of PHLNs, and serum total bilirubin (TBIL) levels 3 months post-KPE were correlated.

**Results:**

The AUC, sensitivity, specificity, PPV, and NPV of PHLNs with hyperechogenicity and a maximum length larger than 8.4 mm were 0.898, 81.8%, 97.8%, 89.6%, and 95.8%, respectively. The combination of PHLNs, TC thickness, and gallbladder morphology achieved the best overall diagnostic efficacy among all indicators with an AUC of 0.927 and a sensitivity of 99.2%. The germinal center number and bile particle number of PHLNs were positively correlated with pathological size and US echogenicity intensity of PHLNs, respectively (*r* = 0.591, 0.377, *p* = 0.001, 0.004). The pathological size of PHLNs in BA patients was negatively correlated with jaundice clearance status 3 months after KPE surgery (*r* = −0.385, *p* = 0.047).

**Conclusion:**

PHLNs with hyperechogenicity and a maximum length > 8.4 mm are useful US indicators for BA diagnosis. Additionally, the enlargement of PHLNs might play a role in predicting outcomes of KPE surgery.

**Critical relevance statement:**

The article proposed for the first time that PHLNs with hyperechogenicity and a maximum length > 8.4 mm are a useful US indicator for diagnosing BA.

**Key Points:**

- PHLNs may be helpful in diagnosing BA and predicting outcomes after surgery.
- Enlarged hyperechoic PHLNs are a useful diagnostic indicator for BA, and play a role in predicting surgical outcomes.
- These findings can assist clinicians in more accurately diagnosing BA, enabling more timely treatments.

**Graphical Abstract:**

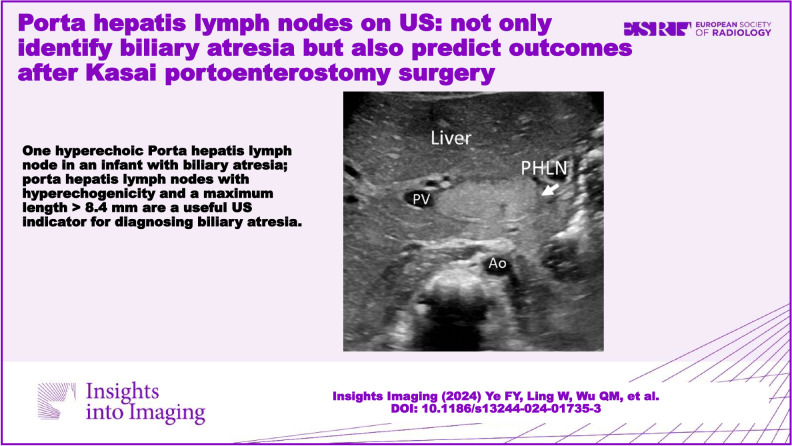

## Introduction

Neonatal cholestasis is a syndrome observed in infants under the age of one, characterized by hepatocellular jaundice, hepatic pathological signs, and liver functional impairment [1]. Common etiologies encompass biliary disorders (such as biliary atresia (BA)), bacterial and viral infections (such as cytomegalovirus), and congenital metabolic abnormalities (such as neonatal intrahepatic cholestasis caused by citrin deficiency (NICCD)), and others. Some cases with unknown causes are referred to as idiopathic neonatal cholestasis.

BA as the prevalent cause of neonatal cholestasis is a progressive, inflammatory, and fibro-obliterative cholangiopathy [2]. The worldwide incidence rate is 5.5–13.0 in 100,000 live patients [3]. Kasai portoenterostomy (KPE), proposed by Kasai in 1959, is still the first-line therapeutic regimen for BA patients [2, 4]. Unlike other etiologies which only require conservative treatment or elective surgery, BA necessitates prompt surgical intervention. Numerous studies indicated that early surgical treatment significantly improves the prognosis of BA. Therefore, an early and accurate diagnosis of BA is crucial for clinical outcomes [2, 5]. There are several diagnostic imaging methods, including ultrasonography (US), hepatobiliary scintigraphy, and magnetic resonance cholangiopancreatography [6–9]. Among them, the US is the most widely used method [10, 11].

Previously, we illustrated that an enlarged porta hepatis lymph node (PHLN) detected on US scans was a useful feature in diagnosing BA [12]. However, this study was only a preliminary study with a small sample and still needs further validation. Moreover, in that study, only the size of the PHLN was regarded as the US diagnostic criterion. After the accumulation of clinical cases, we found that the US features of PHLN not only included the size but also referred to the number, shape, and echogenicity. Thus, the US criterion of PHLNs used for BA diagnosis needs to be updated, which may further improve the diagnostic efficacy of PHLNs.

It has been reported that the pathologic findings of the PHLN at KPE surgery, such as the number of follicular germinal centers, were correlated with the outcomes of KPE surgery [13]. PHLNs with different pathologic findings might have varied US features. Therefore, we assumed that the US features of the PHLNs might not only be useful in diagnosing BA but also helpful in predicting outcomes of KPE surgery.

Thus, the aim of this study was to evaluate the usefulness of PHLNs on US scans in diagnosing BA and predicting the outcomes after KPE surgery in a prospective cohort of patients.

## Materials and methods

### Patients

Between January 2019 and December 2022, data on consecutive patients with neonatal cholestasis were collected in our hospital. The diagnosis of neonatal cholestasis can be made if the serum direct bilirubin (DBIL) value is > 1 mg/dL [1]. This prospective study was approved by the ethics committee of the hospital. Informed consent was obtained from the parents of all patients.

The inclusion criteria were as follows: (1) the patients received US examination and routine serum biomarker examination in our hospital; (2) the patients were less than 5 months of age; (3) the patients were diagnosed as BA or non-BA based on the results of liver biopsy or intraoperative cholangiography (IOC); and (4) the patients were diagnosed as non-BA based on clinical manifestations, and the diagnosis was confirmed by the patient’s recovery from jaundice and normalization of laboratory values during the follow-up period.

### US examination

All patients underwent abdominal US scanning performed by six sonographers, each with 3–16 years of experience in pediatric US, who had received pre-study training. The equipment used was GE Voluson E8/E10 equipped with a 9–11 MHz linear array transducer; GE Logiq E9 equipped with a 9–11 MHz linear array transducer; GE Vivid IQ equipped with an 11 MHz linear array transducer. The patients weren’t fed for at least 4 h before the examination. They were kept quiet and if required, fed with milk during the examination.

Firstly, the porta hepatis region was scanned to determine the presence of PHLN. The PHLNs were located at the porta hepatis, in front of the main portal vein, around the hepatoduodenal ligament. Then the gallbladder fossa was detected. If the gallbladder was detected, its length, lumen, outline, wall, and mucosal lining were evaluated. Finally, the triangular cord (TC) thickness was measured. The TC thickness was defined as the thickness of the hyperechogenic anterior wall of the anterior branch of the right portal vein that was immediately distal to the right portal vein on a longitudinal image without including the branch of the hepatic artery [14]. Both dynamic and static images were stored during the above US examination for each patient. Each diameter was measured at least twice, and the mean value was adopted.

### Routine serum biomarkers

The serum levels of γ-glutamyltransferase (GGT), total bilirubin (TBIL), DBIL, indirect bilirubin (IBIL), alanine transaminase (ALT), and aspartate aminotransferase (AST) within one week of US examination were collected.

### Image analysis

All the dynamic and static images were re-evaluated by two sonographers with 10 years and 16 years of experience in the pediatric US who were blinded to all clinical information.

**Fig. 1:**
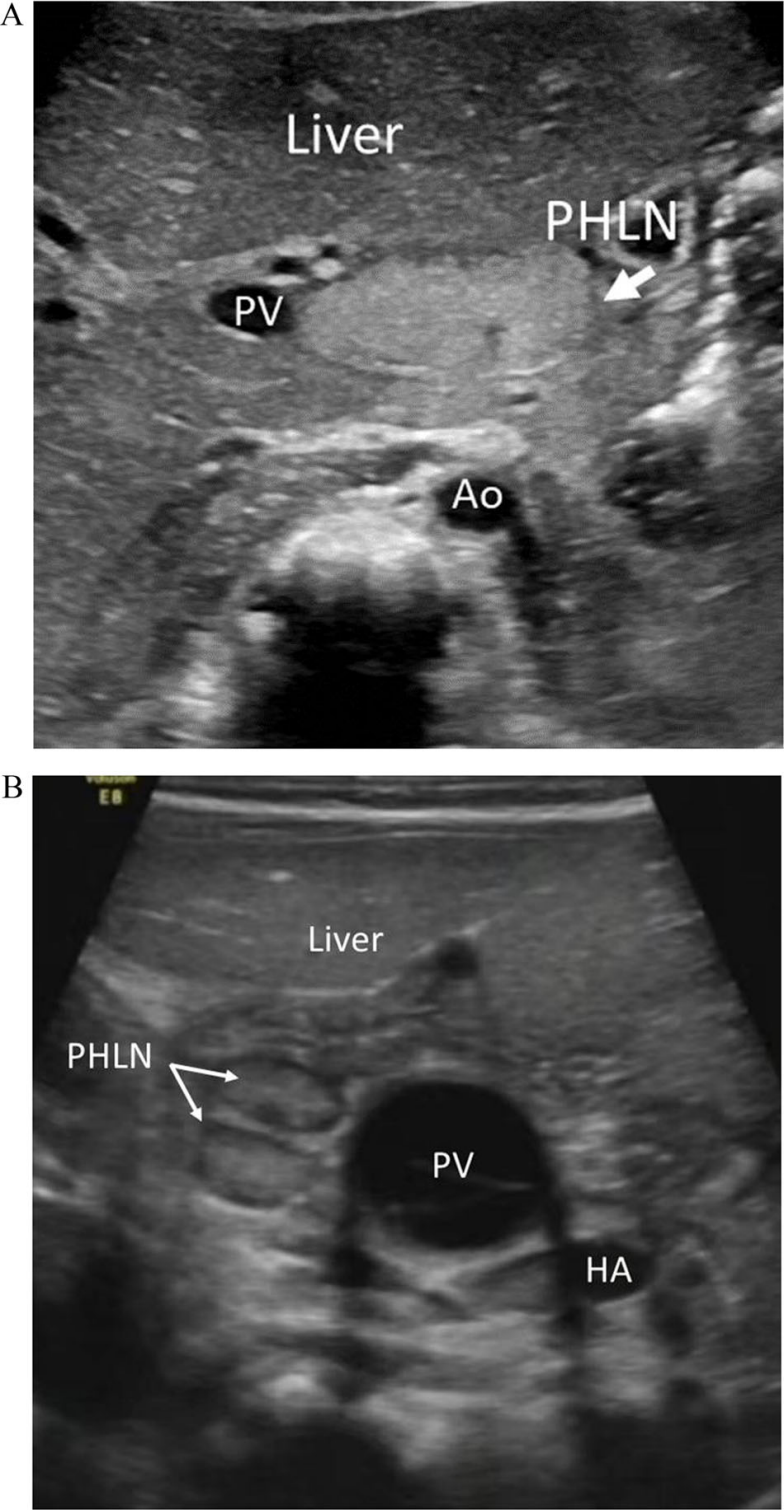
PHLNs at the US examinations. **A** One hyperechoic PHLN with a maximum length of 20.1 mm, maximum width of 8.5 mm, and regular shape in a BA infant. **B** Multiple hypoechoic PHLNs with a maximum length of 7.9 mm, maximum width of 4.0 mm, and regular shape in a non-BA infant. PHLN, porta hepatis lymph node; PV, portal vein; HA, hepatic artery

The maximal length and width of the PHLNs were measured. When the number exceeded two, it was recorded as multiple; otherwise, it was recorded as non-multiple. The shape was categorized as regular or irregular, with regular including circular and elliptical. When the echogenicity of PHLN was higher than that of the liver, the PHLN was considered hyperechoic; conversely, it was considered hypoechoic (Fig. 1). Those with undetectable PHLN were assigned a diameter of zero. If there were multiple PHLNs, only the largest one was used for analysis.

The gallbladder morphology was considered abnormal if at least one of the following criteria was met [14–18]: (1) the gallbladder was absent; (2) the length of the gallbladder was less than 1.5 cm; (3) the outline of the gallbladder was irregular; (4) the wall of the gallbladder was not identifiable, and the gallbladder appeared as a cyst in the gallbladder fossa; (5) the wall thickness of the gallbladder was irregular; and (6) the hyperechogenic mucosal lining of the gallbladder was not smooth or incomplete. The gallbladder morphology was considered normal if one of the following criteria was met: (1) the gallbladder was detected without a lumen, but a smooth and complete hyperechogenic mucosal lining was visualized and the wall was uniformly thickened or the lumen was incompletely filled with a smooth and complete hyperechogenic mucosal lining with the walls in part in close approximation and (2) the gallbladder was detected with a fully filled lumen and lumen length more than 1.5 cm without wall thickening, and the hyperechogenic mucosal lining of the gallbladder was smooth.

The TC thickness was also reviewed, and the cutoff value for TC thickness was determined to be more than 2 mm according to previous studies [14].

### Histopathological analysis

The largest PHLN was chosen as the specimen when a surgical sample included multiple PHLNs. The size of PHLNs was evaluated by counting the cross-sectional area (area = the maximum length × maximum width). The numbers of germinal centers and bile particles of sections under the microscope were counted by two senior pathologists. The bile particles were semi-quantitatively analyzed and classified into three grades: grade 0: no bile particles; grade 1 + : 1–2 bile particles; grade 2 + : 3–10 bile particles; and grade 3 + : > 10 bile particles.

All patients whose PHLN specimens were enrolled were followed up for at least 3 months, and the serum level of TBIL 3 months post-KPE was recorded. In clinical practice, the prognosis of KPE was assessed based on the duration of native liver survival (NLS). Recent studies have demonstrated that serum TBIL < 2 mg/dL at 3 months after KPE was a surrogate predictor for favorable NLS, or in other words, satisfactory KPE prognosis [19–23]. Thus, a serum TBIL level < 2 mg/dL at 3 months after KPE was defined as jaundice clearance status.

### Statistical analysis

The continuous variables were tested for normality by using the Shapiro–Wilk test. The continuous variables were expressed as mean ± standard deviation or median (interquartile range) and compared using an unpaired *t*-test or Mann–Whitney *U*-test according to the normality. The categorical variables were compared using χ^2^ or the Fisher exact test. The combination of indicators was performed according to the rule of the sequential test or parallel test. In the sequential test, the diagnosis was considered positive only when all indicators were positive. In the parallel test, the diagnosis was considered positive if any of the indicators were positive. The diagnostic performance of independent or combined indicators was evaluated by drawing receiver operating characteristic (ROC) curves, and the area under the ROC curve (AUC), sensitivity, specificity, positive predictive value (PPV), and negative prediction value (PPV) were calculated. The comparison of AUCs was performed by the method of the DeLong test.

The correlation between quantitative variables was analyzed by Pearson correlation analysis or Spearman rank correlation analysis. The correlation between the binary variable and the ranking variable was analyzed by Rank-Biserial correlation analysis. The correlation between the binary variable and the quantitative variable was analyzed by Point-Biserial correlation analysis.

The statistical analyses were performed with statistical software SPSS, version 25, and MedCalc Software, version 15.2.2. The statistically significant difference was defined as a *p* value < 0.05.

## Result

### Clinical characteristics

**Fig. 2:**
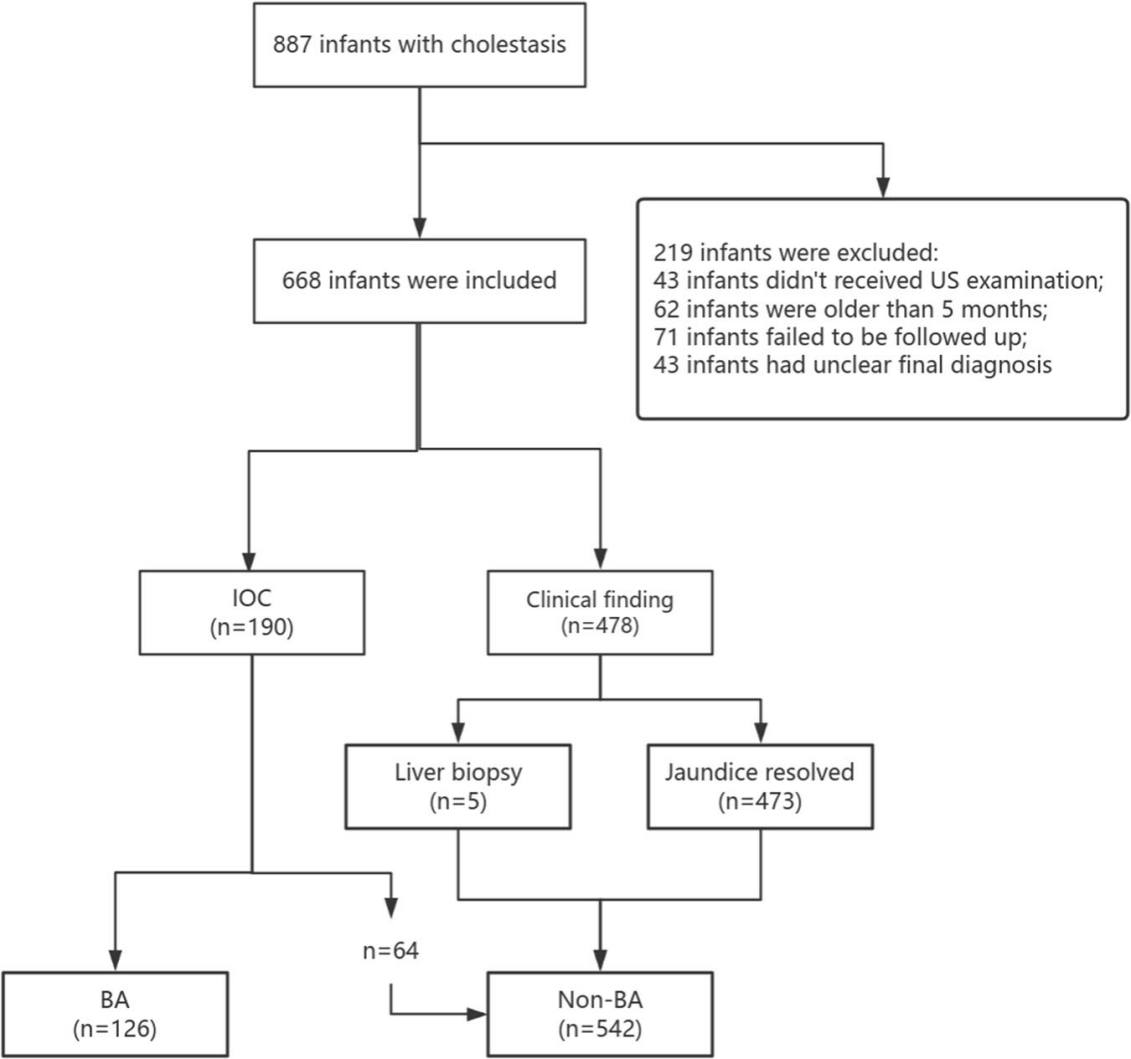
Flow chart of inclusion criteria. IOC, interoperative cholangiography

Data from 887 patients were collected, and 668 patients (126 BA and 542 non-BA) were eligible for inclusion (Fig. 2). There were 431 males and 237 females with a median (interquartile range) age of 49 (34, 69) days. The baseline clinical characteristics of the patients are listed in Table 1. There were differences between BA and non-BA in age, gender, GGT, TBIL, DBIL, IBIL, ALT, and AST (all *p* < 0.01). The diagnosis of 542 non-BA infants is listed in Table 2.

**Table 1:** Clinical characteristics of BA and non-BA infants

	BA (*n* = 126)	Non-BA (*n* = 542)	*p* value
Age (days)	57.0 (48.0, 69.0)	46.0 (32.0, 69.0)	< 0.01
Gender			< 0.01
Female	67	170	
Male	59	372	
GGT (U/L)	535.7 (285.0, 793.3)	144.4 (86.0, 256.4)	< 0.01
TBIL (μmol/L)	157.5 (132.7, 188.5)	102.0 (74.4, 145.9)	< 0.01
DBIL (μmol/L)	115.4 (96.3, 142.0)	72.0 (51.2, 99.4)	< 0.01
IBIL (μmol/L)	40.2 (29.6, 54.1)	29.7 (20.5, 44.5)	< 0.01
ALT (U/L)	128.4 (87.2, 220.7)	72.3 (34.3, 124.7)	< 0.01
AST (U/L)	190.0 (139.3, 280.5)	111.3 (62.8, 188.0)	< 0.01

*GGT* γ-glutamyltransferase, *TBIL* total bilirubin, *DBIL* direct bilirubin, *IBIL* indirect bilirubin, *ALT* alanine transaminase, *AST* aspartate aminotransferase

**Table 2:** Diagnosis of 542 cases of non-BA infants

Diagnosis	Number of cases
Cytomegalovirus infection	287
Rubella virus infection	15
Herpes simplex virus infection	10
Epstein-Barr virus infection	6
Hepatitis B virus infection	5
Syphilis infection	8
Bacterial infection	152
Biliary system stones	1
Choledochal cyst	16
NICCD	18
Progressive familial intrahepatic cholestasis	12
Parenteral nutrition-related cholestasis	5
Tyrosinemia	2
Alagille syndrome	3
Glycogen storage disease	2

### Positive PHLNs

The US characteristics of PHLNs were detailed in the Supplementary Materials. The AUC, sensitivity, specificity, PPV, and Negative predictive value (NPV) of the maximum length of PHLNs were 0.863, 87.3%, 85.2%, 57.9%, and 96.7%. The corresponding values of the maximum width of PHLNs were 0.777, 83.5%, 71.9%, 88.0%, and 63.9%. The AUC of the maximum length was larger than that of the maximum width (*p* = 0.039). Hence, the assessment of PHLN size was based on the maximum length in the following study.

**Fig. 3:**
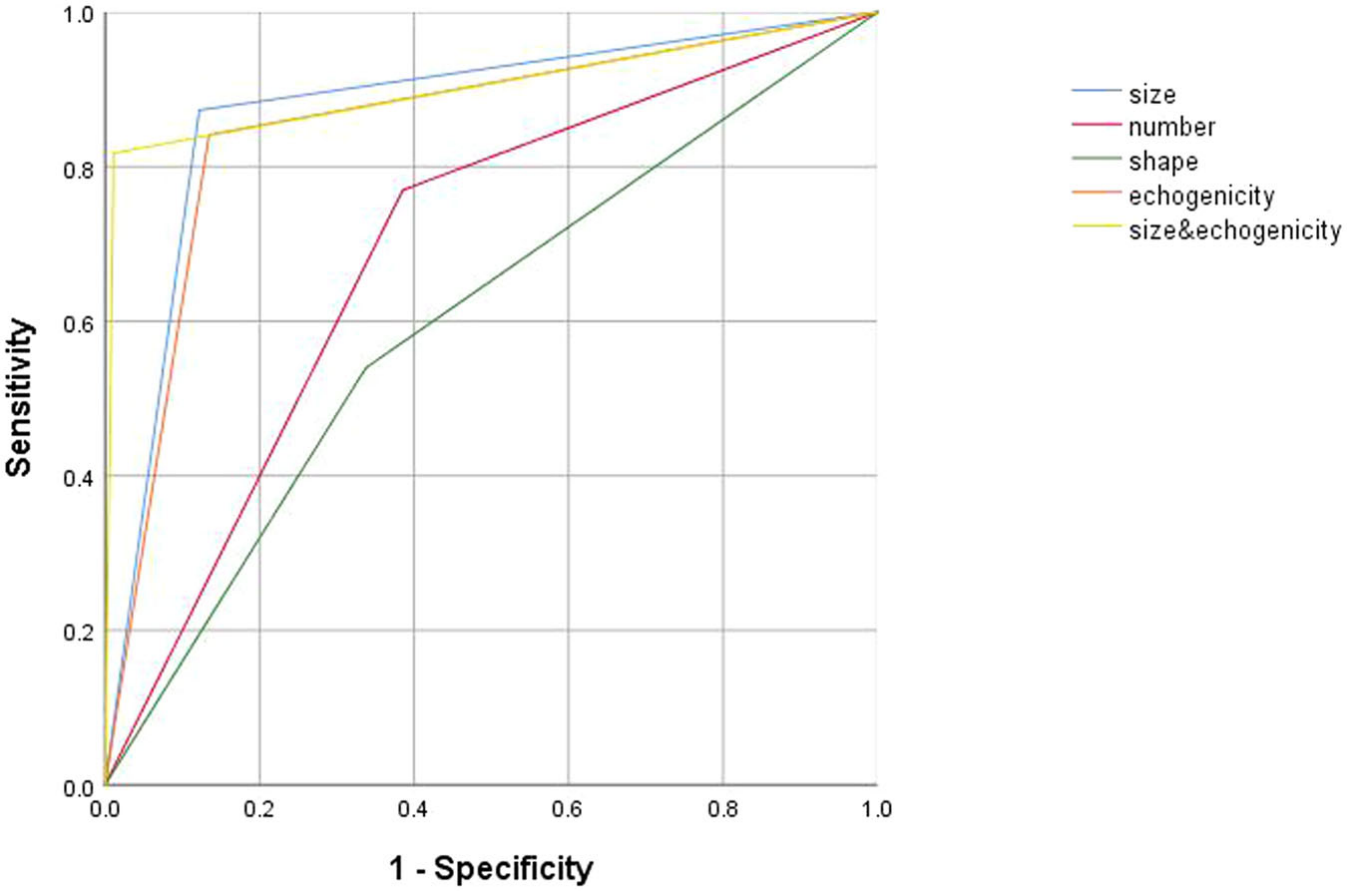
ROC curves for independent and combined indicators of different US features of PHLNs. ROC, receiver operating characteristic; US, ultrasonography; PHLN, porta hepatis lymph node

The independent and combined diagnostic efficacy of PHLN size, number, shape, and echogenicity are listed in Table 3, and the ROC curves are shown in Fig. 3. The combination of indicators was performed according to the rule of the sequential test. Among the independent indicators, the diagnostic efficacy of PHLN size and echogenicity was better than that of the others. Based on the Youden index, the cutoff value of size was set to 8.4 mm of the PHLN maximum length. When the maximum length > 8.4 mm was regarded as BA diagnostic criteria, the corresponding AUC, sensitivity, specificity, PPV, and NPV were 0.863, 87.3%, 85.2%, 57.9%, and 96.7%. When the hyperechoic PHLN was used as the BA diagnostic criterion, the corresponding AUC, sensitivity, specificity, PPV, and NPV were 0.853, 84.1%, 86.5%, 59.2%, and 95.9%.

**Table 3:** The independent and combined diagnostic efficacy of four US indicators of PHLNs

	AUC	Sensitivity (%)	Specificity (%)	PPV (%)	NPV (%)
Size	0.863 (0.834, 0.888)	87.3 (80.2, 92.6)	85.2 (82.0, 88.1)	57.9 (50.5, 65.0)	96.7 (94.6, 98.1)
Number	0.692 (0.656, 0.727)	77.0 (68.6, 84.0)	61.4 (57.2, 65.6)	31.7 (26.5, 37.2)	92.0 (88.7, 94.6)
Shape	0.601 (0.563, 0.638)	54.0 (44.9, 62.9)	66.2 (62.1, 70.2)	27.1 (21.7, 33.0)	86.1 (82.4, 89.3)
Echogenicity	0.853 (0.824, 0.879)	84.1 (76.6, 90.0)	86.5 (83.3, 89.3)	59.2 (51.6, 66.5)	95.9 (93.7, 97.5)
Size and number	0.814 (0.782, 0.843)	74.6 (66.1, 81.9)	88.2 (85.2, 90.8)	59.5 (51.4, 67.2)	93.7 (91.3, 95.7)
Size and shape	0.705 (0.669, 0.740)	51.6 (42.5, 60.6)	89.5 (86.6, 91.9)	53.3 (44.0, 62.4)	88.8 (85.9, 91.3)
Size and echogenicity	0.898 (0.872, 0.920)	81.8 (73.9, 88.1)	97.8 (96.2, 98.9)	89.6 (82.5, 94.5)	95.8 (93.8, 97.3)
Number and shape	0.625 (0.588, 0.662)	50.0 (41.0, 59.0)	75.1 (71.2, 78.7)	31.8 (25.4, 38.8)	86.6 (83.2, 89.5)
Number and echogenicity	0.812 (0.780, 0.841)	73.0 (64.4, 80.5)	89.3 (86.4, 91.8)	61.3 (53.0, 69.2)	93.4 (90.9, 95.4)
Shape and echogenicity	0.724 (0.688, 0.757)	50.8 (41.7, 59.8)	93.9 (91.6, 95.8)	66.0 (55.7, 75.3)	89.1 (86.3, 91.6)
Size, number, and shape	0.697 (0.660, 0.731)	47.6 (38.7, 56.7)	91.7 (89.0, 93.9)	57.1 (47.1, 66.8)	88.3 (85.3, 90.8)
Size, number, and echogenicity	0.843 (0.813, 0.870)	70.6 (61.9, 78.4)	98.0 (96.4, 99.0)	89.0 (81.2, 94.4)	93.5 (91.1, 95.4)
Size, shape, and echogenicity	0.736 (0.700, 0.769)	48.4 (39.4, 57.5)	98.7 (97.4, 99.5)	89.7 (79.9, 95.8)	89.2 (86.4, 91.5)
Number, shape, and echogenicity	0.718 (0.682, 0.752)	47.6 (38.7, 56.7)	95.9 (93.9, 97.4)	73.2 (62.2, 82.4)	88.7 (85.9, 91.2)
Size, number, shape, and echogenicity	0.721 (0.685, 0.754)	45.2 (36.4, 54.3)	98.9 (97.6, 99.6)	90.5 (80.4, 96.4)	88.6 (85.8, 91.0)

*AUC* area under the ROC curve, *PPV* positive predictive value, *NPV* negative predictive value

Among all indicators, the diagnostic efficacy of size combined with echogenicity was highest (Table 3, all *p* < 0.05). Thus, PHLN with both hyperechogenicity and a maximum length > 8.4 mm was regarded as positive PHLN and defined as the BA diagnostic criteria in this study. The AUC, sensitivity, specificity, PPV, and NPV of positive PHLN were 0.898, 81.8%, 97.8%, 89.6%, and 95.8%.

To mitigate potential bias, we conducted subgroup analyses, excluding cases with undetected PHLN and focusing on positive PHLN cases among both BA and non-BA patients attributed to infection. The detailed results of these analyses were provided in the Supplementary Materials.

### TC thickness and gallbladder morphology

The US characteristics of TC thickness and gallbladder morphology in the BA and non-BA groups were detailed in the Supplementary Materials. As Table 4 shows, the AUC, sensitivity, specificity, PPV, and NPV of TC thickness were 0.905, 87.3%, 93.7%, 76.4%, and 96.9%. The corresponding values of gallbladder morphology were 0.889, 87.3%, 90.6%, 68.3%, and 96.8%.

**Table 4:** Independent and combined diagnostic efficacy of three US indicators

	AUC	Sensitivity (%)	Specificity (%)	PPV (%)	NPV (%)
PHLN	0.898 (0.872, 0.920)	81.8 (73.9, 88.1)	97.8 (96.2, 98.9)	89.6 (82.5, 94.5)	95.8 (93.8, 97.3)
GB	0.889 (0.863, 0.912)	87.3 (80.2, 92.6)	90.6 (87.8, 92.9)	68.3 (60.5, 75.4)	96.8 (94.9, 98.2)
TC	0.905 (0.880, 0.926)	87.3 (80.2, 92.6)	93.7 (91.3, 95.6)	76.4 (68.6, 83.1)	96.9 (95.1, 98.2)
PHLN + TC	0.923 (0.900, 0.942)	92.9 (86.9, 96.7)	91.7 (89.0, 93.9)	72.2 (64.7, 79.0)	98.2 (96.7, 99.2)
PHLN + GB	0.934 (0.912, 0.951)	97.6 (93.2, 99.5)	89.1 (86.2, 91.6)	67.6 (60.3, 74.3)	99.4 (98.2, 99.9)
GB + TC	0.925 (0.903, 0.944)	97.6 (93.2, 99.5)	87.5 (84.4, 90.1)	64.4 (57.2, 71.2)	99.4 (98.2, 99.9)
PHLN + GB + TC	0.927 (0.904, 0.945)	99.2 (95.7, 100.0)	86.2 (83.0, 89.0)	62.5 (55.4, 69.2)	99.8 (98.8, 100.0)

*PHLN* porta hepatis lymph node, *GB* gallbladder morphology, *TC* triangular cord, *AUC* area under the ROC curve, *PPV* positive predictive value, *NPV* negative predictive value

### The combined indicators

**Fig. 4:**
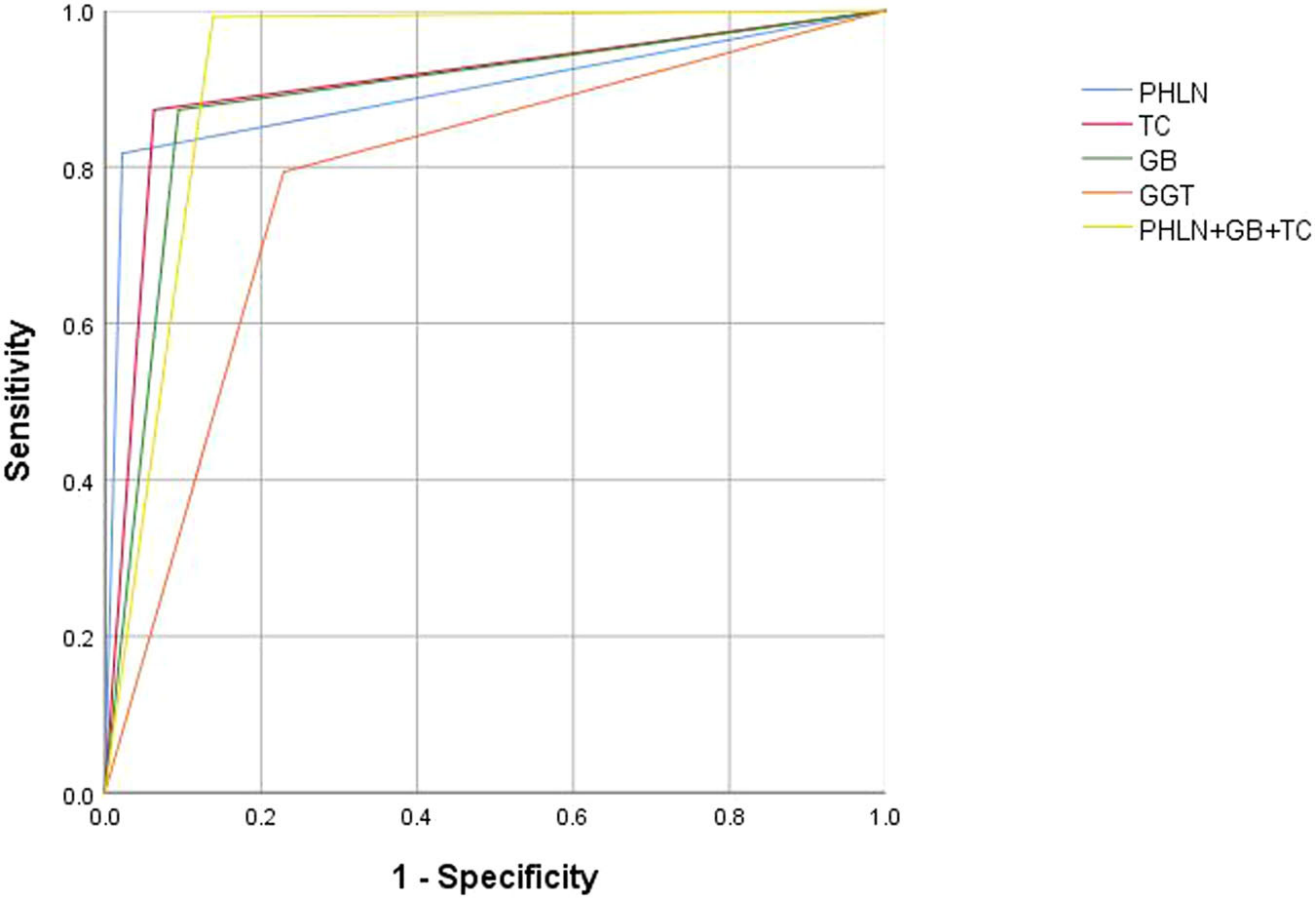
ROC curves of independent and combined indicators of three US indicators. ROC, receiver operating characteristic; US, ultrasonography; PHLN, porta hepatis lymph node

The diagnostic efficacy of the independent and combined indicators is shown in Table 4 and Fig. 4. The combination of indicators was performed according to the rule of the parallel test. The AUCs of gallbladder morphology and TC thickness were improved from 0.889, 0.905 to 0.934, 0.923 when they were combined with PHLNs (all *p* < 0.01). The best two indicators among all independent and combined indicators were PHLN + GB and PHLN + GB + TC. The AUC values of PHLN + GB and PHLN + GB + TC were 0.934, 0.927 and the difference had no statistical significance (*p* = 0.306). While, the sensitivity of PHLN + GB + TC was 99.2%, which was higher than that of PHLN + GB.

### The correlation between histopathological findings and US features of PHLNs in BA patients

Among 126 BA infants who were diagnosed by IOC and underwent KPE surgery, 27 PHLN specimens obtained from 27 patients during surgery were analyzed. The pathological size of the PHLNs was evaluated by counting the cross-sectional area of the specimens. The cross-sectional area of the PHLNs in 27 BA patients ranged from 13.5 mm^2^ to 190.6 mm^2^ (67.8 (39.6, 103.6)) mm^2^.

**Fig. 5:**
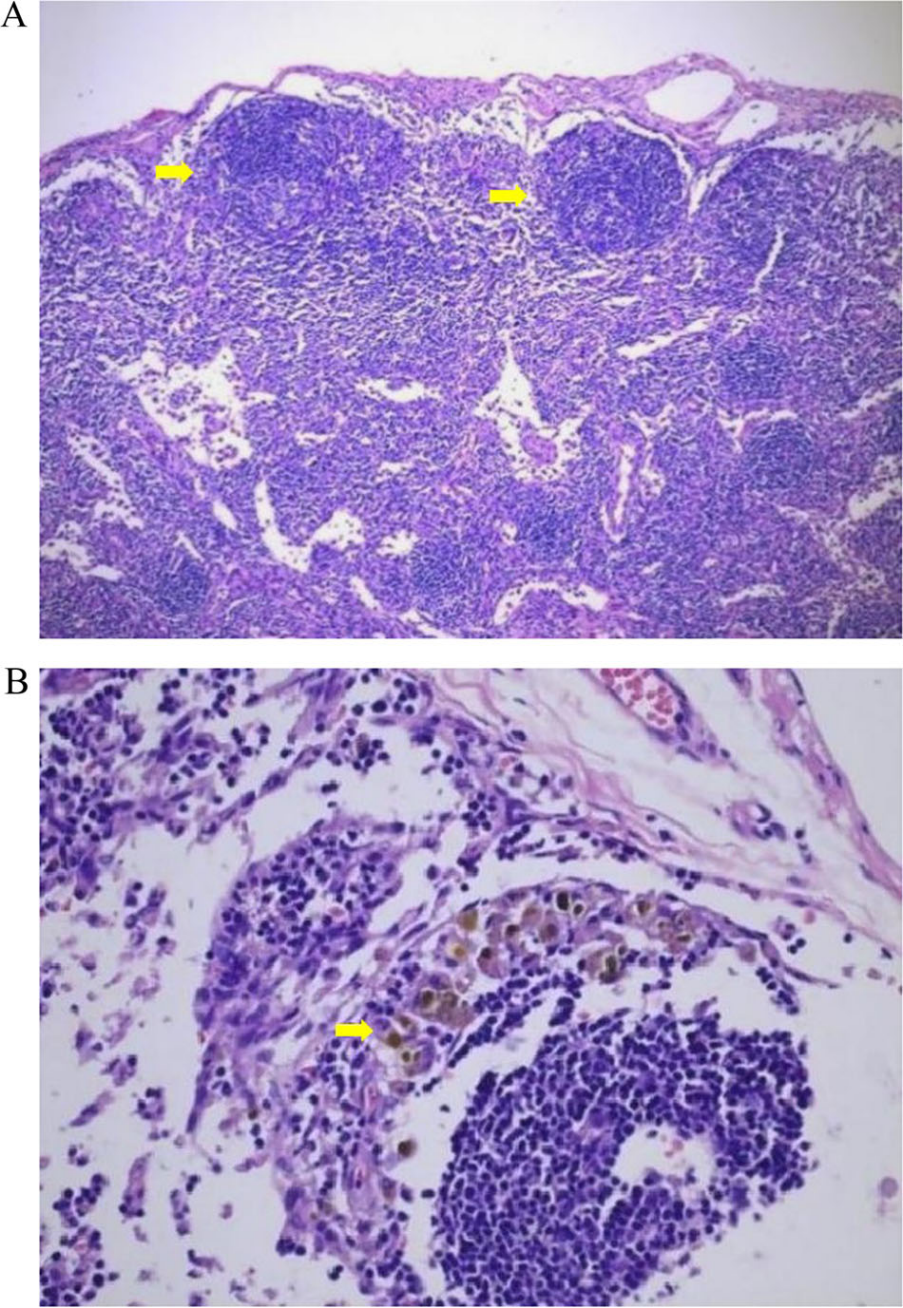
**A** Hematoxylin-eosin staining (HE) of germinal centers in PHLNs (HE × 100), yellow arrows indicated germinal centers. **B** HE staining of bile particles in PHLNs (HE × 400), yellow arrows indicated bile particles. HE, hematoxylin-eosin; PHLN, porta hepatis lymph node

The germinal centers of the PHLN were observed in 25 patients, with a median and interquartile range of 7.0 (4.0, 21.0) (Fig. 5A). There was a positive correlation between the number of germinal centers and the size of PHLNs (*r* = 0.591, *p* = 0.001).

Bile particles were observed in all hematoxylin-eosin (HE) staining sections of the PHLN specimens (Fig. 5B). In the 40× objective lens, the bile particle number of the HE section was counted and graded. Among them, nine patients with 1–2 bile particles were grade 1 + , 7 patients with 3–10 bile particles were grade 2 + , and 11 patients with 3–10 bile particles were grade 3 + . There was a positive correlation between the grade or the number of bile particles and the US echogenicity intensity (*r* = 0.377, *p* = 0.004).

### The correlation between US features of PHLNs and serum TBIL levels 3 months after KPE

The serum TBIL level 3 months after KPE of these 27 infants was collected. A serum TBIL level < 2 mg/dL at 3 months after KPE was defined as jaundice clearance status [19–23]. The pathological size of PHLNs in BA was negatively correlated with jaundice clearance status 3 months after KPE surgery (*r* = −0.385, *p* = 0.047). That is, the larger the pathological area of PHLNs, the worse the status of jaundice clearance 3 months after KPE surgery.

## Discussion

In this study, we demonstrated that PHLNs with hyperechogenicity and a maximum length > 8.4 mm were an effective diagnostic indicator for BA. The diagnostic efficacy of PHLN was close to that of TC thickness and gallbladder morphology. Among all the independent and combined indicators, PHLN + GB + TC achieved the best overall diagnostic efficacy with the highest AUC (0.927) and sensitivity (99.2%). Considering the poor prognosis of BA, this indicator with the highest sensitivity is especially valuable in clinical practice. We also illustrated that the pathological size of PHLNs was positively correlated with the number of germinal centers (*r* = 0.591, *p* < 0.001), and the US echogenicity of PHLNs was positively correlated with the number of bile particles in PHLNs (*r* = 0.377, *p* < 0.004). Moreover, the pathological size of PHLNs was negatively correlated with jaundice clearance status 3 months after KPE surgery (*r* = −0.385, *p* = 0.047).

The mechanism of the enlargement and hyperechogenicity of PHLN is still unclear. Bove proposed that the enlargement of PHLNs might be correlated with germinal centers [13]. In this study, we also verified that the number of germinal centers positively correlated with the size of PHLNs. The germinal center is the site of B-cell differentiation, reflecting the immune response of human lymph nodes to antigen stimulation, which occurs 1–2 weeks after contact with the antigen. As important antigen-presenting cells, macrophages swallow increased bile particles in the liver and then drain into the PHLNs. Macrophages trigger the immune response, leading to reactive proliferation and finally causing the enlargement of PHLNs. The presence of bile particles in PHLNs might also be one of the reasons for the hyperechogenicity of PHLNs on US scans.

TC thickness and gallbladder morphology were the earliest proposed, most important, and most widely used US indicators for BA diagnosis [11, 14, 18, 24, 25]. The AUC of PHLNs was comparable to that of TC thickness and gallbladder morphology (all *p* > 0.05), which implied the value of PHLNs in diagnosing BA. To explore a better indicator, we assessed the diagnostic efficacy of combined indicators based on these three US features. The results showed that the combination of PHLN, TC thickness, and gallbladder morphology could achieve the best overall diagnostic efficacy. Therefore, we proposed that the combination of PHLN, TC thickness, and gallbladder can be an effective US indicator for BA diagnosis.

Clinically, the outcome of KPE is evaluated by the time of NLS. Bove suggested that the reactivity of the PHLNs was correlated with NLS after KPE surgery [13]. We conjectured that the enlargement of PHLNs might reflect the severity of bile duct obstruction. In this study, we found that the pathological size of PHLNs in BA patients was negatively correlated with jaundice clearance status 3 months after KPE surgery (*r* = −0.385, *p* = 0.047). Thus, we assumed that the size of PHLNs pre-KPE can be a predictor of serum TBIL level 3 months after KPE surgery, which means BA patients with larger PHLNs pre-KPE were more likely to achieve shorter NLS and poorer KPE outcomes.

There are some limitations in this study. First, bowel gas can hinder the visualization of the PHLNs, especially those around the pancreas in some infants. In such a situation, we only assess the PHLNs around the main portal vein which can usually be clearly visualized due to its elevated position. This might increase false negative cases. Second, US examination is unavoidably influenced by operators, the absence of an assessment of inter-observer agreement is one limitation of this study. Third, surgeons did not necessarily sample the HPLNs for pathological examination. Therefore, we can currently only provide pathological samples from 27 BA infants. Considering that this is just a small sample preliminary study, its accuracy still needs further investigation in subsequent research.

## Conclusion

PHLNs with hyperechogenicity and a maximum length > 8.4 mm are a useful US indicator for BA diagnosis. Additionally, the enlargement of PHLNs might play a role in the prediction of outcomes of KPE surgery.

### Supplementary information

ELECTRONIC SUPPLEMENTARY MATERIAL

## Data Availability

The data that support the findings of this study are available from the corresponding author, Fengying Ye, upon reasonable request.

## Abbreviations

ALT: Alanine transaminase
AUC: Area under the ROC curve
AST: Aspartate aminotransferase
BA: Biliary atresia
DBIL: Direct bilirubin
GGT: γ-Glutamyltransferase
HE: Hematoxylin-eosin
IBIL: Indirect bilirubin
IOC: Intraoperative cholangiography
KPE: Kasai portoenterostomy
NLS: Native liver survival
NPV: Negative predictive value
NICCD: Neonatal intrahepatic cholestasis caused by citrin deficiency
PHLN: Porta hepatis lymph node
PPV: Positive predictive value
ROC: Receiver operating characteristic
TBIL: Total bilirubin
TC: Triangular cord
US: Ultrasonography
