# Supplementary material for: Porta hepatis lymph nodes on US: not only identify biliary atresia but also predict outcomes after Kasai portoenterostomy surgery
Source: Insights Imaging. 2024 Jun 20;15:154. doi: 10.1186/s13244-024-01735-3 (PMC11189885; doi:10.1186/s13244-024-01735-3)
Supplement: Supplementary file 1 — ELECTRONIC SUPPLEMENTARY MATERIAL [file 13244_2024_1735_MOESM1_ESM.pdf]

Porta hepatis lymph nodes on US: not only identify biliary atresia but also predict outcomes after Kasai portoenterostomy surgery

ELECTRONIC SUPPLEMENTARY MATERIAL

PHLNs

Among the 126 BA patients, PHLNs were present in 119 (94.4%) patients and absent in 7 (5.6%) patients. The maximum length of PHLNs ranged from 6.0 mm to 22.0 mm [9.5 (8.5, 12.5) mm]. The maximum width of PHLNs ranged from 4.0 mm to 16.0 mm [8.5 (7.0, 10.0) mm]. The number of PHLNs was less than or equal to two in 27 (21.4%) patients and more than two in 99 (78.6%) patients. The shape of PHLNs was regular in 51 (42.9%) patients and irregular in 68 (57.1%) patients. The echogenicity was hyperechoic in 106 (89.1%) patients and hypoechoic in 13 (10.9%) patients.

Among the non-BA 542 BA patients, the PHLNs were present in 300 (55.4%) patients and absent in 242 (44.6%) patients. The maximum length of PHLNs ranged from 4.0 mm to 19.5 mm [8.0 (7.1, 8.3) mm]. The maximum width of PHLNs ranged from 3.0 mm to 11.0 mm [4.5 (4.0, 5.5) mm]. The number of PHLNs was less than or equal to two in 333 (61.4%) patients and more than two in 209 (38.6%) patients. The shape of PHLNs was regular in 117 (39.0%) patients and irregular in 183 (61.0%) patients. The echogenicity was hyperechoic in 73 (24.3%) patients and hypoechoic in 227 (75.7%) patients.

Subgroup Analysis excluding cases with undetected PHLN

When PHLNs were not visible, the maximum length was assigned a value of zero. Subgroup analysis of PHLN was conducted to ensure that potential bias resulting from this approach did not compromise the reliability of the results. Subgroup analysis included only 419 cases where visible PHLNs were present. Among these cases, 119 (28.4%) were diagnosed with BA, 306 (73.0%) had multiple PHLNs, 251 (60.1%) exhibited irregular shape, and 180 (42.9%) had hyperechogenicity.

Based on the Youden index, the cut-off value of size was still set to 8.4 mm. As the supplementary Table 1 showed, when the maximum length >8.4 mm was regarded as BA diagnostic criteria, the corresponding AUC, sensitivity, specificity, PPV, NPV were 0.829, 92.4% (110/119), 73.3% (220/300), 57.9% (110/190), 96.1% (220/229). When the hyperechoic PHLN was used as the BA diagnostic criterion, the corresponding AUC, sensitivity, specificity, PPV, NPV were 0.824, 89.1% (106/119), 75.7% (227/300), 59.2% (106/179), 94.6% (227/240). Among all independent and combined indicators, the diagnostic efficacy of size combined with echogenicity was highest (all $P<0.05$), and the corresponding AUC, sensitivity, specificity, PPV, NPV were 0.913, 86.6% (103/119), 96.0% (288/300), 89.6% (103/115), 94.7% (288/304).

Subgroup analysis of positive PHLN between BA patients and non-BA patients attributed to infection

Non-BA group comprised 483 patients attributed to infection (viral or bacterial infection) and 59 patients attributed to non-infection. The subgroup analysis only included 483 non-BA patients attributed to infection and 126 BA patients. The AUC, sensitivity, specificity, PPV, NPV of positive PHLN were 0.897, 81.8%, 97.7%, 90.4%, 95.4%.

TC thickness and gallbladder morphology

Among 126 BA patients, the TC thickness of 110 patients (87.3%) > 2 mm, and the TC thickness of 16 patients (12.7%) ≤2 mm. The TC thickness ranged from 1.2 mm to 6.0 mm [3.0 (2.5, 3.9) mm]. Among the 542 non-BA, the TC thickness of 34 patients (6.2%) > 2 mm, and the TC thickness of 508 patients (93.7%) ≤2 mm. The TC thickness ranged from 1.1 mm to 2.8 mm [0 (0, 0) mm].

Among the 126 BA patients, 110 (87.3%) patients had abnormal gallbladder morphology, and 16 (12.7%) patients had normal gallbladder morphology. Among the 542 non-BA patients, 51 (9.4%) patients had abnormal gallbladder morphology, and 491 (90.6%) patients had normal gallbladder morphology.

Supplementary table 1: The independent and combined diagnostic efficacy of four US indicators of PHLNs

	AUC	Sensitivity(%)	Specificity(%)	PPV(%)	NPV(%)
size	0.829 (0.789%, 0.864)	92.4% (86.1%, 96.5%)	73.3% (67.9%, 78.3%)	57.9% (50.5%, 65.0%)	96.1% (92.7%, 98.2%)
number	0.559 (0.510, 0.607)	81.5% (73.4%, 88.0%)	30.3% (25.2%, 35.9%)	31.7% (26.5%, 37.2%)	80.5% (72.0%, 87.4%)
Shape	0.519 (0.470, 0.568)	42.9% (33.8%, 52.3%)	61.0% (55.2%, 66.6%)	30.4% (23.5%, 37.9%)	72.9% (67.0%, 78.3%)
echogenicity	0.824 (0.784, 0.859)	89.1% (82.0%, 94.1%)	75.7% (70.4%, 80.4%)	59.2% (51.6%, 66.5%)	94.6% (90.9%, 97.1%)
size&number	0.788 (0.746, 0.826)	79.0% (70.6%, 85.9%)	78.7% (73.6%, 83.2%)	59.5% (51.4%, 67.2%)	90.4% (86.2%, 93.7%)
size&shape	0.678 (0.631, 0.723)	54.6% (45.2%, 63.8%)	81.0% (76.1%, 85.3%)	53.3% (44.0%, 62.4%)	81.8% (77.0%, 86.0%)

size& echogenicity	0.913 (0.882, 0.938)	86.6% (79.1%, 92.1%)	96.0% (93.1%, 97.9%)	89.6% (82.5%, 94.5%)	94.7% (91.6%, 97.0%)
number& shape	0.540 (0.491, 0.588)	52.9% (43.6%, 62.2%)	55.0% (49.2%, 60.7%)	31.8% (25.4%, 38.8%)	74.7% (68.4%, 80.3%)
number& echogenicity	0.790 (0.748, 0.828)	77.3% (68.7%, 84.5%)	80.7% (75.7%, 85.0%)	61.3% (53.0%, 69.2%)	90.0% (85.7%, 93.3%)
shape& echogenicity	0.714 (0.668, 0.757)	53.8% (44.4%, 63.0%)	89.0% (84.9%, 92.3%)	66.0% (55.7%, 75.3%)	82.9% (78.4%, 86.9%)
size&number& shape	0.677 (0.630, 0.722)	50.4% (41.1%, 59.7%)	85.0% (80.4%, 88.8%)	57.1% (47.1%, 66.8%)	81.2% (76.4%, 85.4%)
size&number& echogenicity	0.856 (0.818, 0.888)	74.8% (66.0%, 82.3%)	96.3% (93.5%, 98.2%)	89.0% (81.2%, 94.4%)	90.6% (86.8%, 93.6%)
size&shape& echogenicity	0.745 (0.700, 0.786)	51.3% (41.9%, 60.5%)	97.7% (95.3%, 99.1%)	89.7% (79.9%, 95.8%)	83.5% (79.2%, 87.2%)
number&shape	0.715	50.4%	92.7%	73.2%	82.5%

&echogenicity	(0.670, 0.758)	(41.1%, 59.7%)	(89.1%, 95.3%)	(62.2%, 82.4%)	(78.0%, 86.4%)
size&number& shape& echogenicity	0.729 (0.684, 0.771)	47.9% (38.7%, 57.2%)	98.0% (95.7%, 99.3%)	90.5% (80.4%, 96.4%)	82.6% (78.2%, 86.4%)
